# Bovine viral diarrhea virus 2 strains generate deletion viral genomes primarily in the NS2 region of the viral genome

**DOI:** 10.3389/fvets.2025.1686098

**Published:** 2025-09-25

**Authors:** David J. Holthausen, Darrell O. Bayles, John D. Neill, Rohana P. Dassanayake, Shollie M. Falkenberg, Daniel W. Nielsen, Anna K. Goldkamp, Harish Menghwar, Eduardo Casas

**Affiliations:** ^1^Ruminant Diseases and Immunology Research Unit, National Animal Disease Center, USDA, Agricultural Research Service, Ames, IA, United States; ^2^Infectious Bacterial Diseases Research Unit, National Animal Disease Center, USDA, Agricultural Research Service, Ames, IA, United States; ^3^Department of Pathobiology, Animal Health Research, College of Veterinary Medicine, Auburn University, Auburn, AL, United States; ^4^ARS Research Participation Program, Oak Ridge Institute for Science and Education (ORISE), Oak Ridge, TN, United States

**Keywords:** bovine viral diarrhea virus 2, pestivirus, non-standard viral genomes, deletion viral genomes, viral evolution

## Abstract

Bovine viral diarrhea virus (BVDV) is a significant economic concern for the global cattle industry. This is attributed to the increased risk of transplacental BVDV infection during pregnancy, before sufficient maturation of the fetal immune system. This results in significant reproductive losses via spontaneous abortion as well as the birth of offspring that are persistently infected and immunotolerant to the non-cytopathic BVDV strain. These persistently infected cattle act as reservoirs and are the major source of BVDV transmission within the herd. Previously, we reported bioinformatic analyses showing that BVDV1a and 1b genotypes generate distinct sets of diverse deletion viral genomes (DelVGs) during the natural viral life cycle. DelVGs are generated by skipping events that occur during genome synthesis by the error-prone viral replication machinery. These replication-deficient genomes play many roles in host–pathogen interactions, contributing to the establishment of viral persistence. A total of 21 field isolates of the BVDV2 genotype were analyzed for the presence and characterization of DelVGs using Illumina MiSeq BVDV2 genome sequencing reads. BVDV2 strains generate significantly more Nonstructural protein 2 (NS2) DelVGs than any other region of the genome, with over 90% of those deletions having both 5` and 3` junctions within the NS2 region. BVDV2c strains produce approximately 150 times as many DelVG reads as BVDV2a strains. Of the BVDV2a isolates queried, cytopathic BVDV2a strains generated two times as many NS2 DelVG reads as compared to non-cytopathic BVDV2a strains.

## Introduction

Bovine viral diarrhea virus (BVDV) is a positive-sense single-stranded RNA virus of the *Pestivirus* genus of the *Flaviviridae* family. BVDV causes substantial economic losses in the cattle industry due to reproductive failure and is a known contributor to the bovine respiratory disease complex, which presents as bacterial and viral pneumonia (1–6). BVDV immunosuppresses the host, predisposing the animal to secondary infections and worse symptoms. Most consequentially, transplacental infection often results in abortion, resorption, or the generation of persistently infected (PI) calves. PI calves arise when a transplacental non-cytopathic (NCP) BVDV infection occurs during approximately 25–125 days of gestation when the fetal immune response is in an immature state that allows for tolerization to the virus but not termination of the fetus or clearance of the virus (2, 7–9).

*Pestivirus* is a genus of 11 viral species that primarily infect mammals, including major livestock species such as *Bos taurus, Sus scrofa domesticus, and Bison bison* (10). The approximately 12.3-kilobase BVDV genome is encoded as a single open reading frame that is post-translationally cleaved, lacks a 5` cap and poly-A tail, and encodes 5` and 3` untranslated regions (UTRs). The genome encodes four structural proteins, including a capsid/core (C) protein and three envelope (E) glycoproteins—E1, E2, and a unique to pestivirus, E^rns^ protein. The non-structural proteins (NS) encoded include the unique N-terminal autoprotease NPro, p7, NS2-3 proteases, NS4A, NS5A, and the RNA-dependent RNA polymerase NS5B (10–14). BVDV exhibits high genetic diversity, generating quasispecies. It is currently divided into two genotypes or species, BVDV1 and BVDV2, and 25 subgenotypes, BVDV1a–u and BVDV2a–d, respectively (15–17). Two biotypes of BVDV exist, cytopathic (CP) and NCP. Only NCP viral strains establish persistent infections during transplacental infection (18). In addition to quasispecies, we recently reported that BVDV1a and BVDV1b strains generate diverse sets of deletion viral genomes (19). This was the first reporting of DelVGs being generated by a pestivirus.

DelVGs are a form of non-standard or defective viral genome (NSVG/DVG). NSVGs are a naturally occurring phenomenon in both positive- and negative-sense RNA viruses. During viral replication, genomic variants are generated with internal truncations that render the nascent genome deficient in self-propagation without the presence of a helper standard virus (20). Although the mechanism of DelVG generation is an area of active research, and many proteins are involved, the RNA-dependent RNA polymerase and its intrinsic error-prone nature and varying degrees of fidelity are central to this process. During replication, DelVGs are generated when the polymerase separates from the RNA genome, skips over a region, then reassociates downstream of the separation point, and continues to generate the now truncated daughter strand (20, 21). DelVGs are packaged into defective interfering particles (DIPs) and are known to modulate virulence by competing with standard functional viral genomes for replication machinery, stimulating the interferon response, and aiding in the establishment of persistence (20–26). BVDV1a and BVDV1b genotypes generate distinct sets of DelVGs, with BVDV1a strains generating significantly more C region deletions than BVDV1b strains (19). The study reports that BVDV2 strains, including subgenotypes BVDV2a, 2b, and 2c, produce their own unique profiles of DelVGs.

## Materials and methods

### Virus field isolates and RNA extraction

A total of 21 BVDV2 field isolates (16 BVDV2a, 1 BVDV2b, and 4 BVDV2c) obtained from clinical samples submitted to the National Animal Disease Center were selected for this analysis. BVDV2 strains were isolated from serum, buffy coat, or tissue homogenate samples (27). These samples were inoculated onto Madin–Darby bovine kidney (MDBK) cells in minimal essential medium (MEM) [Gibco MEM 11095 supplemented with 10% filtered and heat-inactivated BVDV antigen-free and BVDV antibody-free fetal bovine serum (HI-FBS)] for 1 h at 37 °C and 5% CO_2_. The inoculum was washed off the cells, and fresh media containing 10% BVDV and BVDV antibody-free FBS was added to the cells before returning to 37 °C and 5% CO_2_ for 4–5 days prior to collection. In the absence of cytopathic effect (CPE), cells were fixed and stained (19, 28). Viral RNA was extracted using QiaCube Viral RNA kits (Qiagen, Valencia, CA, USA) according to the manufacturer’s instructions (29). Genomic sequences of BVDV2a, BVDV2b, and BVDV2c were obtained from NCBI GenBank as references for phylogenetic comparison.

### Determination of cytopathic effect

Whether a BVDV strain is CP or NCP is determined by the visualization of CPE that alters cell morphology upon infection in cell culture. Confluent plates of MDBK and bovine turbinate (BTu) cells in a 12-well plate were inoculated with 200 μL of each viral isolate or cell control and rocked on a shaker for 1 h at 37 °C. MEM (Gibco MEM 11095 supplemented with 10% filtered and HI-FBS) was added to each well and incubated at 37 °C and 5% CO_2_. The presence or absence of CPE was checked at 24-, 48-, 72-, 96-, and 120-h post-inoculation. The biotype was confirmed at the 120-h time point.

### Whole-genome sequencing and phylogenetic analyses

For whole-genome sequencing, cDNA was synthesized from viral RNA using 20-base primers of known sequence with eight random bases at the 3`-end of the primer to produce barcode-identifiable cDNAs, which were then amplified via primer-specific polymerase chain reaction (27). Sequencing was completed using the MiSeq platform (Illumina, Inc., San Diego, CA, USA). BVDV genomes were assembled via reference-assisted and *de novo* assembly using SeqManNGen Version 12 (LaserGene, Inc., Madison, WI, USA). The initial classification was completed by aligning the 5` UTR sequences of the assembled viruses, as is common practice (28, 30). Phylogenetic analysis for relatedness within the BVDV2 genotype was conducted by alignment of the entire genome. The Molecular Evolutionary Genetics Analysis version 12 (MEGA12- iGEM, Temple University, Philadelphia, PA, USA) software’s ClustalW alignment tool and UPGMA method were used to generate phylogenetic dendrograms with branch support estimated via 1,000 bootstrap replicates and the Poisson correction method to calculate evolutionary distance (31).

### Viral Opensource DVG Key Algorithm 2 (VODKA2)

The Viral Opensource DVG Key Algorithm 2 (VODKA2) bioinformatic pipeline was conducted in the local runtime environment (32). The VODKA2 workflow requires a specified reference sequence to analyze the DelVGs. For this study of BVDV2, we used the McCart_c complete genome (NCBI GenBank MH806438.1) as the reference genome. The genome was formatted into a VODKA2-specific large Bowtie2 database via the “genomefa_to_newfasta_del_v2.pl” VODKA2 script using the following parameters: length of the reference genome in base pairs (bp), sequencing read length of 151 bp, and gap size of 10 bp. After generating the Bowtie2 database, the paired-end Illumina MiSeq reads for each field isolate genome were analyzed against the reference database via the VODKA2_analysis_setup.sh script. Deletion viral genome “species” consists of reads with the same theoretical deletion size but with 5` and 3` junctions within a range of ±5 nucleotides. For each field isolate sample analyzed, DelVG “species” with only a single (non-normalized) read were excluded from the analysis. “Read coverage” denotes the normalization of DelVG reads per one million standard viral reads sequenced. The comparison of normalized non-standard reads has been previously described in the literature (33).

### Statistical analyses

Statistical analyses were performed via GraphPad Prism (GraphPad Software, Inc. Version 10.2.0, Boston, MA, USA). For the analysis of deletion species size and DelVG read coverage per species across viral isolates, two-way ANOVA with Tukey’s multiple comparison test was performed. For analysis of deletion read coverage per strain across subgenotype and biotype, unpaired *t*-test was performed. For comparison of 5` and 3` junction read coverage per viral strain across subgenotype and biotype, two-way ANOVA with Ŝídák’s multiple comparison test was performed. For analysis of deletion read coverage per strain, delineated by biotype and subgenotype, an ordinary one-way ANOVA with Tukey’s multiple comparison test was performed.

## Results

### BVDV2 strains generate different quantities and sizes of DelVGs

**Figure 1:**
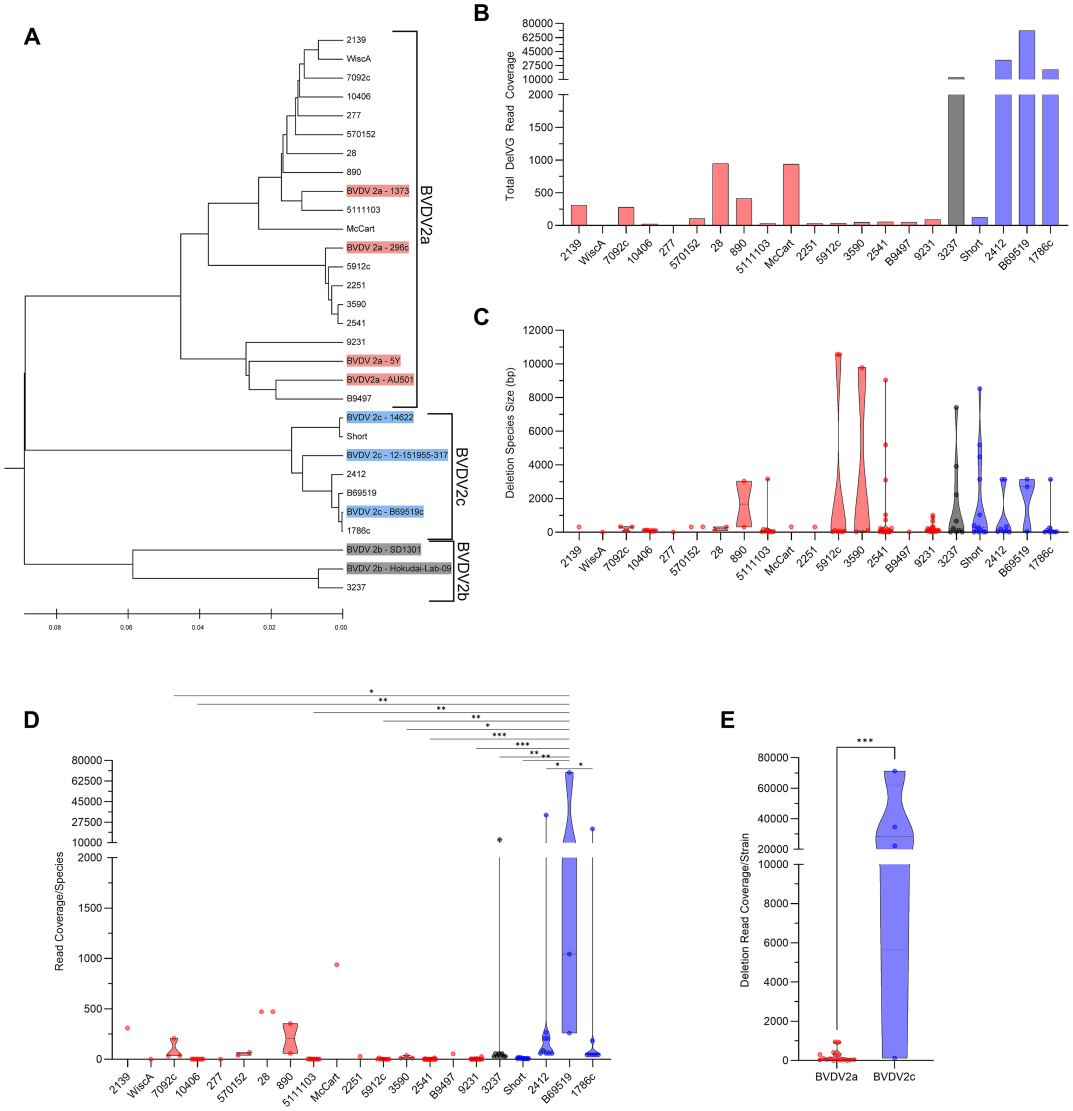
Phylogenetic DelVG analysis of BVDV2 strains. **(A)** Phylogenetic dendrogram of BVDV2 strains. Comparative genomes for subgenotyping are highlighted. BVDV2a (Red), BVDV2b (Gray), BVDV2c (Blue). **(B)** Total strain DelVG read coverage per 1 million standard viral reads. BVDV2 strains are ordered along the x-axis by full-genome ClustalW phylogenetic analysis using MEGA12 software. BVDV2a (red), BVDV2b (Gray), and BVDV2c (Blue). **(C)** Strain-specific DelVG species sizes (bp). BVDV2 strains are ordered along the x-axis by full-genome ClustalW phylogenetic analysis using MEGA12 software. BVDV2a (Red), BVDV2b (gray), and BVDV2c (Blue). There were no significant differences. **(D)** Strain-specific read coverage per DelVG species per 1 million standard viral reads. BVDV2 strains are ordered along the x-axis by full-genome ClustalW phylogenetic analysis using MEGA12 software. BVDV2a (red), BVDV2b (Gray), and BVDV2c (Blue). Ns: *p* > 0.05, *: *p* < 0.05, **: *p* < 0.005, ***: *p* < 0.0005. **(E)** Total deletion reads (read coverage per 1 million standard viral reads) among BVDV2 strains. ***: *p* < 0.0005.

To characterize the profile of DelVGs generated by BVDV2 strains, 21 BVDV2 viral isolates were selected for analysis. Of these, 15 strains were previously sequenced (27, 34, 35). The viral isolates were mapped against a known BVDV2 genome to confirm sequence fidelity ahead of VODKA2 NSVG analysis. The phylogenetic relationship between the analyzed strains and subgenotypes was confirmed by whole-genome sequence alignment of all experimental strains with verified BVDV2a, 2b, and 2c sequences obtained from GenBank. 16 of the selected experimental isolates were identified as BVDV2a strains, one was BVDV2b, and four were BVDV2c by phylogenetic analysis (Figure 1A). A full list of GenBank accession numbers for experimental and comparative viral strains is presented in Supplementary Table 1. Of the 21 BVDV2 isolates, 19 generated DelVG species. Only BVDV2a strains WiscA and 277 did not generate DelVGs (Figure 1B). There were no significant differences in the generated DelVG species size among BVDV2 strains (Figure 1C). Read coverages for the DelVG species were variable across BVDV2 strains. The major deletion species in BVDV2c strains was a particularly predominant DelVG generated by virus B69519, exhibiting significantly greater read coverage than that observed in 11 other BVDV2 strains (Figure 1D). BVDV2c strains generated an average of 32,034 deletion reads per million standard viral reads, whereas BVDV2a strains generated only 210 deletion reads per million standard viral reads per strain (Figure 1E).

### BVDV 2a and 2c DelVG hotspots are primarily generated in the NS2 region

**Figure 2:**
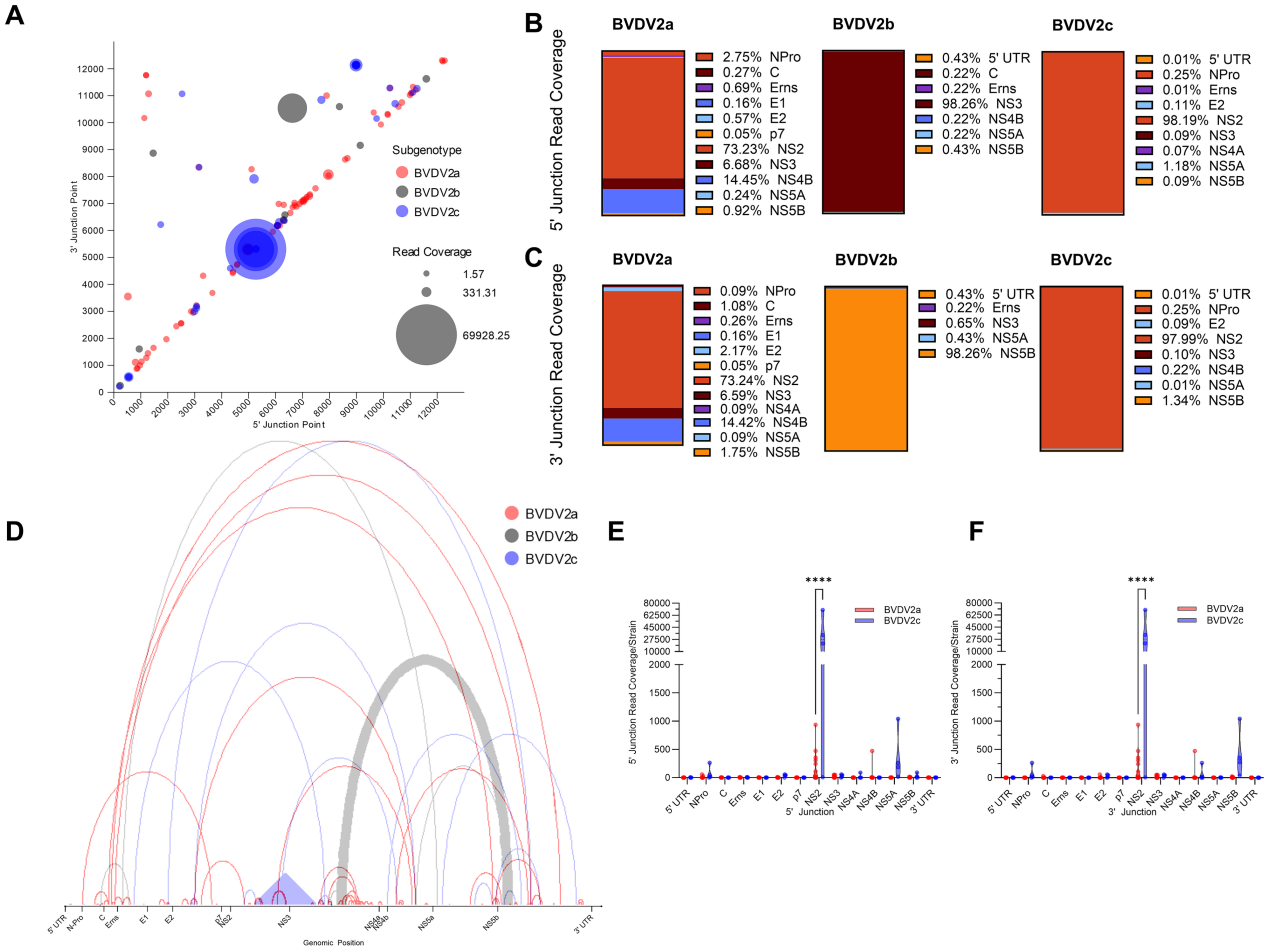
BVDV2 DelVG species and regional hotspots. **(A)** 5` and 3` junction point analysis for all BVDV2 strains. The area of each data point correlates to the read coverage per 1 million standard viral reads. Distribution of 5` DelVG junction points **(B)** and 3` DelVG junction points **(C)** per gene feature for BVDV2a, BVDV2b, and BVDV2c strains. **(D)** Representative depiction of the 5` and 3` junction points of individual DelVGs. Each arc represents an individual DelVG species. The line thickness of each arc is proportional to read coverage (per 1 million standard viral reads) per DelVG species. Locations of the 5` **(E)** and 3` **(F)** junction points of BVDV2a and BVDV2c DelVG reads (read coverage per 1 million standard viral reads. Ns: *p* > 0.05, ****: *p* < 0.0001).

BVDV2 isolates generate DelVGs across the entire length of the genome. For BVDV2 strains, especially BVDV2c strains, there is a strong hotspot in the 5,000–6,000-bp region. The sole BVDV2b strain analyzed had an additional hotspot for a 5` junction at approximately 7,000-bp, and a 3` junction at approximately 10,000-bp (Figure 2A). The DelVG hotspots observed along the BVDV2 genome primarily occur in the NS2 region of the viral genome. For BVDV2a and BVDV2c strains, this translates to approximately 73% of 5` and 3` junctions for 2a and 98% of 5` and 3` junctions for 2c, respectively. The only other region with greater than 10% of DelVG reads for either BVDV2a or 2c was approximately 14% of BVDV2a reads corresponding to deletions in the NS4B region (Figures 2B–D). Within the BVDV2a set, three common deletions were observed in at least two strains, with two (NS2-NS2 4,978–5,296 and NS3-NS3 6,072–6,187) found in five viral isolates each (33%). Within the BVDV2c set, three common deletions were observed (NS2-NS2 5,266–5,306, NS5A-NS5B 8,988–12,135, and NPro-NPro 548–566). The NS2–NS2 5,266–5,306 DelVG was observed in three of four isolates (75%) and accounted for 99.5% of NS2 DelVGs in the set. A NS5A-NS5B 8,988–12,135 DelVG was present in all four BVDV2c isolates (100%), and an NPro-NPro 548–566 was also present in three of four (75%) BVDV2c isolates (Table 1). The single BVDV2b isolate queried had no NS2 DelVG junctions. The DelVG species generated by the BVDV2b strain, accounting for 98.26% of reads, had a 5` junction point in the NS3 region and 3` junction point in the NS5B region (Figures 2B–D). Observing where 5` and 3` junctions occur for BVDV2a and 2c subgenotypes (BVDV2b was excluded from this comparison for only having a single isolate), we found that the increase in DelVGs generated by BVDV2c strains in comparison to BVDV2a is attributable to the amount of NS2 deletions generated by BVDV2c strains (Figures 2E,F). The majority of BVDV2a DelVG reads were in the NS2 region. Four BVDV2c strains generated significantly greater DelVG read coverage in the NS2 region than BVDV2a isolates.

**Table 1:** Most common deletion species (*n* ≥ 2 strains).

Feature 5′-3′ junctions	Number of strains present in	5′ Junction point (bp locus)	3′ Junction point (bp locus)	Deletion size (bp)	Read coverage
BVDV2a (*n* = 16)					
NS2–NS2	5	4,978	5,296	318	1,413
NS2–NS2	2	4,978	5,300	322	979
NS3–NS3	5	6,072	6,187	115	72
BVDV2c (*n* = 4)					
NS2–NS2	3	5,266	5,306	40	168,997
NS5A–NS5B	4	8,988	12,135	3,147	1,814
NPro–NPro	3	548	566	18	529

Common DelVG species were determined by the presence in two or more of the BVDV2a and BVDV2c analyzed strains. From left to right: gene feature where 5` and 3` junctions occur; Number of strains DelVG species is present in; 5` junction point base pair locus in genome; 3` junction point base pair locus in genome; size of DelVG in base pairs; sum of read coverage across all strains present, normalized per 1 million standard viral reads.

### BVDV2a cytopathic strains generate more NS2 deletions than non-cytopathic strains

**Figure 3:**
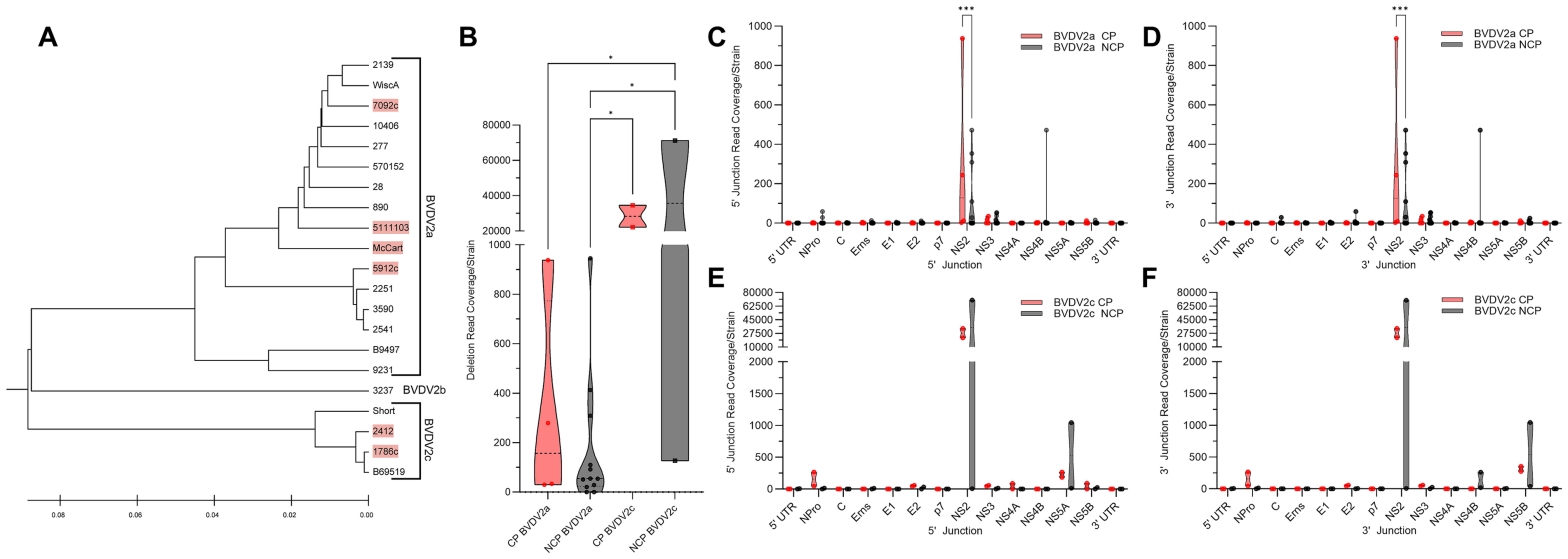
Comparative analysis of BVDV2 biotype DelVGs. **(A)** Phylogenetic dendrogram of BVDV2 strains. Cytopathic strains highlighted in red. **(B)** Total deletion reads (read coverage per 1 million standard viral reads) among BVDV2a and BVDV2c CP and NCP strains. Ns: *p* > 0.05, *: *p* < 0.05. Locations of the 5` **(C)** and 3` **(D)** junction points of BVDV2a CP and NCP DelVG reads (Read coverage per 1 million standard viral reads). Ns: *p* > 0.05, ***: *p* < 0.0005. Locations of the 5` **(E)** and 3` **(F)** junction points of BVDV2c CP and NCP DelVG reads (read coverage per 1 million standard viral reads). There were no significant differences.

We sought to distinguish whether any of the differences in DelVG generation or species characterization were correlated with biotype. From the dual infection of MDBK and BTu cells, four of 16 (25%) BVDV2a strains analyzed were of the CP biotype, and two of four (50%) BVDV2c strains were of the CP biotype. The remaining 15 strains, including the single BVDV2b strain, did not elicit CPE (Figure 3A). Comparing the total read coverage of BVDV2a and BVDV2c CP and NCP strains, we observed that the BVDV2c NCP strains generated more DelVGs than either BVDV2a CP or NCP strains, and BVDV2c CP strains generated more DelVGs than BVDV2a NCP strains (Figure 3B). Comparing where junctions occur between biotypes, BVDV2a CP strains generate greater DelVG read coverage in the NS2 region than NCP strains for both 5` and 3` junctions (Figures 3C,D). In contrast, for BVDV2c strains, there was no difference between CP and NCP strains in the amount and location of 5` and 3` junction read coverage (Figures 3E,F).

## Discussion

BVDV is a virus with substantial genomic diversity across its two genotypes and 25 subgenotypes (16). Recently, the first identification and characterization of DelVGs by BVDV or any other pestivirus was reported (19). Holthausen et al. (19) focused on the most prevalent and closely related genotypes, BVDV1a and BVDV1b. However, it was a small subset of BVDV viral diversity. This study sought to continue this analysis of BVDV DelVGs in BVDV2 with the goal of characterizing deletion profiles to better understand their potential roles in pestivirus biology and reveal new targets for intervention strategies.

Here, the DelVGs generated by 16 BVDV2a, one BVDV2b, and four BVDV2c strains were assessed. An acutely different profile than that of BVDV1 viral strains was found. These differences included unique hotspots for deletions. For the 41 BVDV1a strains, hotspots for deletions were observed in the 900-, 4,500-, and 9,000-bp regions, which correlated with the C, NS2, and NS5A genome regions. For 33 BVDV1b strains, a 5` junction hotspot at 8,000 bp and 3` junction hotspot at 9,000 bp were identified, which correlated with NS4B and NS5A regions (19). In contrast, for BVDV2 strains, there was a strong hotspot in the 5,000–6,000-bp region, especially for BVDV2c strains, which correlated with the NS2 and NS3 genome regions. Unlike BVDV2a and BVDV2c strains, the sole BVDV2b strain analyzed had a hotspot for the 5` junction at approximately 7,000 bp in the NS3 region and the 3` junction at approximately 10,000 bp in the NS5B region. The analysis of BVDV1a vs. BVDV1b found that 52% of DelVGs were generated in the C region by BVDV1a strains, in contrast to 8% in BVDV1b strains. The profile among the BVDV2 strains queried differed greatly. NS2 and NS3 deletions comprise the majority (90%) of BVDV2 DelVG reads, in contrast to 52% of BVDV1a DelVG reads being in the C region and 34% of BVDV1b DelVG reads being NS4B–NS5A deletions. Research into other virus species in *Flaviviridae* have demonstrated that viruses with deletions within the C region can still generate and package recoverable virus, however with varying levels of attenuation (36–38). Future research is needed to determine why these profiles differ and what the implications are for BVDV replication dynamics and pathogenesis. The reduction in C deletions seen in BVDV2 strains and BVDV1b strains in comparison to BVDV1a strains suggests an evolutionary advantage to not producing C deletion genomes, as circulating BVDV1a strains have reduced in comparison to other genotypes (39, 40). The reduced immune induction likely afforded by BVDV1a strains from generating these C deletion genomes does not outweigh the attenuation, making alternative deletions, such as the NS2 and NS3 deletions found in BVDV2 strains, more advantageous.

Unlike the C protein that is critical for viral structure and assembly, NS2 is not a structural element of the virus. The BVDV NS2 protein has cysteine autoprotease functionality (41, 42). As an autoprotease, NS2 self-cleavage of separate NS2 and NS3 proteins from the NS2–NS3 precursor of the BVDV polyprotein is critical for the generation of infectious viruses, viral replication, and virion morphogenesis in CP strains (43). Unlike the C protein, there is no reporting of internal NS2 or NS3 deletions producing recoverable attenuated virus. The NS2 region accounted for 73% of BVDV2a and 98% of BVDV2c DelVG read coverage. One caveat of the NS2 function is that the effective cleavage of the NS2-3 junction by NS2 is linked with the determination of biotype (41). CP BVDV strains are associated with the functional cleavage of the NS3 product, whereas NCP BVDV strains generate uncleaved NS2–NS3 (44, 45). When CP and NCP virus strains were compared among queried BVDV2 isolates, a significant difference was observed between BVDV2a strains for NS2 DelVGs, with CP viruses generating more NS2 DelVGs. As DelVGs are known to stimulate the interferon response, these NS2 DelVGs may be acting in a cytopathogenic accessory role. In the small sample size (*n* = 4) of BVDV2c strains, no difference was observed. Whether a larger set of BVDV2c strains would produce differences between CP and NCP viruses requires further investigation. Holthausen et al. (19) found that no difference was observed between paired CP and NCP viruses for BVDV1 strains; however, NS2 deletions were not the predominant species generated by those strains. As only infection with NCP BVDV strains generates PI animals, the difference in NS2 DelVGs between CP and NCP BVDV2a strains may impact infection-derived cellular immune and survival signaling pathways that shape virulence and pathogenesis. Whether PI-derived BVDV generates unique DelVG profiles and what role those genomes have on viral fitness and persistence should be studied further.

For the sole BVDV2b strain examined, 98% of DelVG reads had a 5` junction in NS3 and a 3` junction in NS5B. The NS3 protein has two domains with separate serine protease and RNA helicase functionalities, which cleave the viral polyprotein and aid in RNA viral genome replication, respectively (46–48). Beyond cleavage in NS3, this DelVG species generates genomes that delete the entirety of NS4A, a cofactor for NS3 activity; NS4B, an integral membrane protein with viral replication and immune evasion activity; and NS5A, a phosphoprotein critical for viral replication, with the 3` cleavage occurring in the RNA-dependent RNA polymerase (13). DelVGs are packaged into DIPs, replacing full-length functional viral genomes in newly generated viral particles. As DelVGs are shorter than standard length viral genomes, they are faster and more efficient to replicate (25, 49, 50). This predominant DelVG generated by the BVDV2b strain deletes most of the non-structural proteins of BVDV and produces a genome that is 32% (3,915 bp) shorter than the standard viral genome, which likely replicates in a more efficient manner than the standard-length virus. The main functions of DelVGs and DIPs during infection are to sequester replication machinery from the standard virus, interfere with standard viral replication, stimulate the interferon response, and promote cellular pro-survival signals that stimulate viral persistence (20, 25, 26). While this predominant DelVG species in the BVDV2b strain could be an anomaly among 2b strains, and further analysis of isolates is needed, the pattern of NS2 deletions among the 20 BVDV2a and 2c isolates suggests that generating these DelVGs is beneficial to the virus.

Single-point mutations in the BVDV NS2 protein are associated with viral attenuation, viability, and cytopathogenicity (51, 52). Based on the prevalence of NS2 DelVGs among BVDV2 strains observed in this study, they likely play a substantive role in BVDV2 viral pathogenesis, moderating viral infection and shaping host cellular responses. Given that DelVGs have been associated with influencing virulence, the establishment of viral persistence, and interferon stimulation in other members of *Flaviviridae* (20, 22, 24, 26, 53), hotspots for viral DelVG generation such as the C region in BVDV1 and now the reporting of NS2 for BVDV2 in this study become targets of interest for understanding virus–host interactions, viral evolution, as well as antiviral and vaccine development.

## Data Availability

The datasets presented in this study can be found in online repositories. The names of the repository/repositories and accession number(s) can be found in the article/Supplementary material.
