# Supplementary material for: Bovine viral diarrhea virus 2 strains generate deletion viral genomes primarily in the NS2 region of the viral genome
Source: Front Vet Sci. 2025 Sep 25;12:1686098. doi: 10.3389/fvets.2025.1686098 (PMC12507611; doi:10.3389/fvets.2025.1686098)
Supplement: Supplementary file 1 [file Table_1.docx]

| **Supplemental Table 1. BVDV GenBank Accession Numbers** | | | | |
| --- | --- | --- | --- | --- |
| **Isolate Name** | **BVDV Subgenotype** | **Year of Isolation** | **Country-Region** | **GenBank Accession #** |
| 28 | 2A | 2016 | USA-AL | MH231141 |
| 277 | 2A | 1993 | USA | PX122060 |
| 890 | 2A | 1990 | USA-IA | U18059 |
| 2139 | 2A | 1992 | USA-IA | MH231125 |
| 2251 | 2A | Unknown | USA | PX122061 |
| 2412 | 2C | 1989 | USA | MH231152 |
| 2541 | 2A | Unknown | USA | PX122062 |
| 3237 | 2B | 1990 | USA | MH231126 |
| 3590 | 2A | 1989 | USA | PX122063 |
| 9231 | 2A | 2004 | USA-OK | MH806437 |
| 10406 | 2A | 1993 | USA-SD | MH231123 |
| 570152 | 2A | 1992 | USA-IA | MH231128 |
| 5111103 | 2A | 2005 | USA-OK | PX122064 |
| 1786c | 2C | 1989 | USA | MH231124 |
| 53637c | 2A | 2003 | Canada | MH231127 |
| 5912c | 2A | 1995 | USA-MO | MH231129 |
| 7092c | 2A | 1989 | USA | PX122065 |
| B69519c | 2C | 2006 | USA | MH231133 |
| B9497 | 2A | 1997 | USA-WY | MH231134 |
| McCart_c | 2A | 1989 | USA | MH806438 |
| Short | 2C | 1989 | USA | MH231149 |
| WiscA | 2A | 1991 | USA-WI | MH231146 |
| *12-151955-317* | *2C* | *2012* | *USA* | *MH231150* |
| *1373* | *2A* | *Not Listed* | *Not Listed* | *AF145967* |
| *14622* | *2C* | *2005* | *USA* | *MH231151* |
| *296c* | *2A* | *1995* | *USA* | *MH806436* |
| *5Y* | *2A* | *2005* | *USA-SD* | *KT875152* |
| *AU501* | *2A* | *2006* | *USA* | *MH231131* |
| *B69519c* | *2C* | *2006* | *USA* | *MH231133* |
| *SD1301* | *2B* | *2012* | *China* | *KJ000672* |
| *Hokudai-Lab-09* | *2B* | *2010* | *Japan* | *AB567658* |
| *GenBank Verified Sequences for Comparative Analysis* | | |  |  |
